# Clinical Software and Bad Decisions: The “Practice Fusion” Settlement and Its Implications

**DOI:** 10.1007/s11673-022-10187-7

**Published:** 2022-04-11

**Authors:** Megan Prictor

**Affiliations:** 1 Melbourne Law School, The University of Melbourne, Parkville, VIC 3010 Australia; 2 Centre for Digital Transformation of Health, The University of Melbourne, Parkville, VIC 3010 Australia

**Keywords:** Whistle-blowing, Pharmaceutical companies, Clinical decision support software, Opioid prescribing, Kickbacks

This article describes a recent United States (U.S.) government settlement with a company producing clinical decision support software (CDSS) for kickbacks the company received from a pharmaceutical company intended to drive up opioid prescribing. It reflects on the legal avenues pursued by the government in the matter and considers the implications of the case for regulation of clinical software design in the Australian healthcare context.

CDSS is medical software that is widely used in both primary care and hospital settings. It is usually integrated within electronic health record (EHR) systems. The software applies algorithms to patient data to generate personalized guidance for a patient’s care that can be utilized by a clinician. CDSS can provide support and reminders to clinicians for decisions about preventative healthcare tasks, prescribing, diagnostic imaging, pathology testing, and many other aspects of a person’s treatment. These systems have been available for over three decades, and there is substantial evidence demonstrating their generally positive effects on care quality, processes, and outcomes.

The regulation of CDSS in Australia is somewhat of a grey zone. I will return to this point after outlining the U.S. *Practice Fusion* case since the case highlights the risks of under-regulation in this arena, particularly in terms of the risks and responsibility it creates for individual clinicians using EHR systems.

## Facts

In January 2020, a settlement was announced in the matter of the *United States of America v. Practice Fusion*. Practice Fusion is a company (now owned by Allscripts) established in 2005 that develops an EHR software platform and supplies it to medical clinics (www.practicefusion.com). It claims to be the leading cloud-based ambulatory EHR platform in the country. At the time of the unlawful activity, it was providing its EHR (and sometimes also computers) to doctors for free, deriving revenue from advertising directed at doctors.

From 2013 onwards, Practice Fusion solicited and received US$1 million in kickbacks from a pharmaceutical company, later identified as Purdue Pharma. The payments were for creating an alert in the EHR designed to increase the prescription of extended-release opioid medication (and hence the sale of Purdue’s products) to treat patients’ pain symptoms. The court heard that Purdue Pharma’s marketing staff helped to design the software alert, which ignored evidence-based clinical guidelines for patients with chronic pain. The alert presented opioid prescription as an option on the same footing with other, evidence-based options such as exercise, cognitive behavioural therapy, and non-opioid analgesics for pain. The alert was triggered in clinical practices some 230 million times between 2016 and 2019 and resulted in additional prescriptions of extended-release opioids numbering in the tens of thousands, causing untold human harm (United States of America v Practice Fusion, Inc 2020, ¶114). Most of the prescriptions were paid for by federal healthcare programmes (United States of America v Practice Fusion, Inc 2020, ¶116).

A U.S. government investigation uncovered this fraud, which occurred on the back of separate unlawful conduct by Practice Fusion relating to having falsely obtained government certification for its software. The company had concealed a failure to comply with certain certification requirements such as data portability and the use of standardized vocabularies. This false certification in turn led to software users inadvertently falsely claiming government incentive payments by attesting that the software they were using complied with government requirements, when in fact it did not.

In early 2020, an investigation conducted by the U.S. Attorney and the Department of Health and Human Services resulted in Practice Fusion admitting to a criminal conspiracy (with Purdue Pharma) and a criminal kickback. The company reached a settlement agreement involving the payment of some $145 million in civil and criminal fines. Individual staff of the company were also pursued for obstruction of the investigation. Purdue was subject to fines in the billions of dollars and criminal forfeiture for this and other unlawful activity, with the U.S. Attorney stating “Purdue’s drug marketers paid to invade the sanctity of the physician-patient relationship so that it could influence medical decisions and increase prescriptions of its most potent opioids” (United States Attorney’s Office, District of Vermont 2020). Importantly, the settlement agreement refers to, although it does not name, other companies that also paid to influence the development and implementation of CDS alerts to increase sales of their pharmaceutical products. There is, therefore, no reason to anticipate that the Purdue/Practice Fusion conspiracy was an isolated incident within the sphere of clinical software development.

## U.S. Legal Avenues

Practice Fusion was pursued under the U.S. Criminal Code 18 U.S.C. § 371 relating to the criminal conspiracy and also under the Anti-Kickback Statute (AKS) (42 U.S.C.), a 1972 law designed to “remove the corrupting effects of kickbacks in health care by outlawing behaviour designed to game the system” (Fader et al. 2020, 5). AKS § 1320a-7b(b)(1)(B) states:

**(b) I** **llegal remunerations**

**(1)** Whoever knowingly and willfully solicits or receives any remuneration (including any kickback, bribe, or rebate) directly or indirectly, overtly or covertly, in cash or in kind—

**…**

**(B)** in return for purchasing, leasing, ordering, or arranging for or recommending purchasing, leasing, or ordering any good, facility, service, or item for which payment may be made in whole or in part under a Federal health care program,

shall be guilty of a felony and upon conviction thereof, shall be fined not more than $100,000 or imprisoned for not more than 10 years, or both.

It is unclear how the unlawful behaviour came to light. However, in similar instances in the United States, the involvement of a whistle-blower had proved to be key. In an article on the regulation of EHRs, Kenagy describes major fraud investigations of four medical software vendors by the U.S. government in the past decade (including Practice Fusion) and the legal avenues the government utilized to identify the wrongdoing and punish the offenders (Kenagy 2021). Importantly, at least two of the four matters commenced with *qui tam* whistle-blower actions, which are available in the United States whereby a person (a “relator”) brings an action on the government’s behalf. The plaintiff is the government and if the action succeeds the relator receives a portion of the award. Under the False Claims Act (USC § 3729) covering fraudulent claims and false records, the relator receives up to 30 per cent of the award. Faunce and colleagues have previously described this and a wide range of other statutory tools available to the U.S. State and Federal governments to recover fraudulently made healthcare claims (Faunce et al. 2010).

## Legal Remedies in Other Countries: Australia as an Example

Other countries need to consider how they will identify and regulate these hidden conflicts and financial inducements whereby unethical and unlawful promotion of certain pharmaceuticals could occur without users knowing. To take one example, in Australia, diverse legal pathways are also available to penalize *Practice Fusion*-style wrongdoing. For instance, fraud can be prosecuted by the Commonwealth Director of Public Prosecutions under the *Criminal Code Act 1995* (Cth), such as s 134.2 which punishes with up to ten years’ imprisonment the offence of obtaining a financial advantage from a Commonwealth entity by deception. The Victorian *Crimes Act 1958* s 176 (and similar legislation in other Australian states) makes it an offence to receive or solicit a secret commission, also punishable by up to ten years’ imprisonment. The misleading and deceptive conduct provisions (s 18) of the *Australian Consumer Law* could enable the Australian Competition and Consumer Commission (ACCC) to act against an EHR vendor if the latter had represented to purchasers that a CDSS’s alerts were wholly evidence-based. The Therapeutic Goods Administration (TGA) could pursue the vendor for failure to comply with the Essential Principles, which include that the medical device “will not compromise the clinical condition or safety of a patient” (Principle 1)—particularly if it were shown that harm or injury had resulted from such non-compliance (*Therapeutic Goods Act 1989* (Cth) ss 41MA, 41MAA).

The risk of fraudulent activities in Australia in relation to EHR design cannot be rejected as fanciful, particularly as the financial conditions are similar, with government incentives for clinics’ use of digital health products and heavy subsidies for pharmaceutical products under the Pharmaceutical Benefits Scheme. To be clear, there has already been some attention paid to pharmaceutical advertising embedded in clinical software in Australia (for example Harvey et al. 2005), but, to my knowledge, none yet in regard to less overt influences on software design in relation to clinical recommendations. Nor it is apparent how such influences might be uncovered. Certainly, clinicians might raise concerns with the TGA about the content of clinical software and with the ACCC about false advertising. Uncovering the type of kickback scheme that existed between Purdue Pharma and Practice Fusion, were one to occur, may be more challenging. The absence of *qui tam* provisions in Australia means that the incentive for a whistle-blower is primarily a moral, certainly not an economic, one. Corporate sector whistle-blowers are protected from victimization or having their identity disclosed, under Part 9.4AAA of the *Corporations Act 2001* (Cth), but they do not receive a share of any award in a claim brought by government that is instigated by their whistle-blowing. There have been repeated calls for the introduction of *qui tam* provisions in Australia including from the Australian Federal Police and organizations such as Civil Liberties Australia. Several opportunities to do this have emerged over the past decade, including the potential introduction of legislation by independent senator Nick Xenophon in 2013 and as part of the Treasury review of tax and corporate whistle-blower protections (2016–17), but none has been taken up.

One opportunity clearly presents itself to improve the transparency of pharmaceutical company funding of EHR companies in Australia. Medicines Australia—an umbrella organization representing the pharmaceutical industry—already voluntarily discloses pharmaceutical company funding of healthcare providers, consumer organizations, and funding for events under its Code of Conduct. It would be useful if any contributions to software vendors influencing the design of EHRs marketed in Australia were an additional area of disclosure by Medicines Australia.

Taking a broader view, the overall “light-touch” regulatory approach to CDSS, premised on prioritizing the clinician’s responsibility for decisions about the application of decision guidance to individual patients, is problematic. The Royal Australian College of General Practitioners has called for an overarching body to be created to oversee the development and maintenance of technical and clinical standards for CDSS tools and recommends that the software content should be based on current, evidence-based guidelines. This is an important and welcome step, since otherwise the burden of identifying inappropriate recommendations appears to fall—heavily—upon the shoulders of individual clinicians.

## Conclusion

The *Practice Fusion* case shines a spotlight on the potential for CDSSs to be vehicles for fraud, leading to widespread harm. The tools for uncovering such fraud and harm in Australia are less robust than in the United States. Tightening both self-regulation by the pharmaceutical industry, for example a Medicines Australia-style self-reporting scheme in relation to medical software, and external oversight of CDSS’ standards, is warranted.
